# Treatment of closed femoral shaft fractures in children aged 2–10 years: a systematic review and meta-analysis

**DOI:** 10.1007/s00068-021-01752-7

**Published:** 2021-08-02

**Authors:** Stijn van Cruchten, Eefke C. Warmerdam, Dagmar R. J. Kempink, Victor A. de Ridder

**Affiliations:** 1 UMC Utrecht, Heidelberglaan 100, 3584 CX Utrecht, The Netherlands; 2 Reinier Haga Orthopedisch Centrum, Toneellaan 2, 2725 NA Zoetermeer, The Netherlands; 3 Erasmus MC/Sophia Children’s Hospital, Wytemaweg 80, 3015 CN Rotterdam, The Netherlands; 4 Kromme Nieuwegracht 15, 3512 HC Utrecht, The Netherlands

**Keywords:** Femur shaft fractures, Pediatric, Intramedullary nails, Spica cast, Traction, Titanium elastic nails

## Abstract

**Objective:**

To review current literature on treatment of closed femoral shaft fractures in children of 2–10 years old, with subgroup analysis of children aged 2–6 years, comparing intramedullary nailing (IMN) to conservative treatment modalities.

**Methods:**

We included clinical trials and observational studies that compared traction and subsequent casting (TSC), spica casting and IMN for treatment of femur shaft fractures in children of 2–10 years of age. Subgroup analysis of children aged 2–6 years was performed.

**Results:**

Compared to treatment with immediate spica casting, IMN led to significantly less coronal angulation (mean difference (MD): 2.03 degrees, confidence interval (CI) 1.15–2.90), less sagittal angulation (MD: 1.59 degrees, CI 0.82–2.35) and lower rates of LLD (Risk difference (RD): 0.07, CI 0.03–0.11). In terms of rehabilitation, IMN leaded to shorter time until walking with aids (MD: 31.53 days, CI 16.02–47.03), shorter time until independent ambulation (MD: 26.59 days, CI 22.07, 31.11) and shorter time until full weight bearing (MD: 27.05 days, CI 6.11, 47,99). Compared to TSC, IMN led to a lower rate of malunion (RD: 0.31, CI 0.05–0.56), shorter hospital stays (MD: 12.48 days, CI 11.57, 13.39), time until walking with aids (MD: 54.55, CI 40.05–69.04) and full weight bearing (MD: 27.05 days [6.11, 47,99]).

**Conclusion:**

Although a lack of quality evidence, this systematic review showed a clear tendency to treatment with elastic intramedullary nails of femoral shaft fractures in children of 2–10 years of age.

**Level of evidence:**

3.

## Introduction

Despite a multitude of treatment options being available, femoral shaft fractures in children continue to pose a challenge to trauma and orthopedic surgeons. These fractures are only seen in 1.4% [1] to 1.7% [2] of all pediatric fractures, and usually lead to lengthy hospitalization, prolonged periods of disability and may cause asymmetry in skeletal growth [3, 4].

A multitude of treatment options have been described for these fractures. Both conservative options such as traction and spica casting and surgical options as elastic intramedullary nailing, plate fixation or a lateral femoral nail are used in daily practice. According to current consensus guidelines, treatment should differ according to age; younger children are advised to be treated with traction and/or spica cast while surgical intervention is preferred in older children [5, 6]. Although the choice of treatment method in pediatric femur fractures in all age groups can be challenging, this is particularly difficult in children between 2 and 10 years of age: no consensus exists on whether conservative or surgical treatment is the best option for this particular age group.

We hypothesized that intramedullary nails (IMN) may be the superior treatment option for children aged 2–10 years. Therefore, this systematic review aims to critically appraise the current literature on treatment of femoral shaft fractures in children of 2–10 years old and to perform subgroup analysis for children of 2–6 years old.

## Methods

This study was conducted by following the PRISMA guidelines. This review did not require approval from the independent ethics committee or institutional review board of the participating institutions.

### Search strategy and selection criteria

To identify relevant literature on the treatment of pediatric femoral shaft fractures, we performed a systematic literature search on Pubmed, Embase and Cochrane. Databases were searched from inception to August 15th, 2020. As most common treatment modalities, we included traction with and without subsequent spica casting, immediate spica casting and intramedullary nailing. In the final search, the following keywords and their synonyms were used: “femoral shaft fractures”, “pediatric”, “conservative”, “cast”, “traction” and “intramedullary nail” A complete clinical query and search are depicted in Tables 1, 2. Our search strategy was finetuned with backward reference searching.

**Table 1:** Components of literature search

	Domain: Femoral shaft fractures in children of 2–6 years old		Determinant: Conservative and surgical treatments of femoral shaft fractures	Outcome: Radiological outcome, rehabilitation, costs
Search term	Femoral shaft fractures	Children	Traction, Intramedullary Nail, Spica Cast	-
Synonyms	Femoral shaft fracture* Femur fracture*	Pediatric, Paediatric Children Child Infan* Toddler* Minor Minors* Boy Boys Girl Girls Kid Kids Schoolschild* Juvenil* Prematur* Youth Youths	Conservative Nail* Titanium Nailing Intramedullary Intra-medullary Casting Casts	-
Keywords	“Femoral fractures” [Mesh]	“Child, preschool” [Mesh] “Child” [Mesh]	“Fracture fixation” [Mesh] “Traction” [Mesh]	-

**Table 2:** Final searches

Pubmed	("Fracture fixation"[MeSH Terms] OR "Traction"[MeSH Terms] OR "conservative"[Title/Abstract] OR "nail*"[Title/Abstract] OR "cast"[Title/Abstract] OR "casting"[Title/Abstract] OR "casts"[Title/Abstract] OR "Traction"[Title/Abstract] OR "intramedullary"[Title/Abstract] OR "intra-medullary"[Title/Abstract] OR "nailing"[Title/Abstract] OR "titanium"[Title/Abstract]) AND ("child, preschool"[MeSH Terms] OR "paediatric"[Title/Abstract] OR "pediatric"[Title/Abstract] OR "children"[Title/Abstract] OR "infan*"[Title/Abstract] OR "toddler*"[Title/Abstract] OR "minor"[Title/Abstract] OR "minors*"[Title/Abstract] OR "boy"[Title/Abstract] OR "boys"[Title/Abstract] OR "girl"[Title/Abstract] OR "girls"[Title/Abstract] OR "kid"[Title/Abstract] OR "kids"[Title/Abstract] OR "schoolchild*"[Title/Abstract] OR "juvenil*"[Title/Abstract] OR "prematur*"[Title/Abstract] OR "youth"[Title/Abstract] OR "youths"[Title/Abstract] OR "child"[MeSH Terms] OR "child"[Title/Abstract]) AND ("femoral shaft fracture*"[Title/Abstract] OR "femoral fractures"[MeSH Terms] OR "femur fracture*"[Title/Abstract]) →2207 results
Embase	('fracture fixation'/exp OR 'traction therapy'/exp OR 'conservative':ab,ti,kw OR nail*:ab,ti,kw OR 'cast':ab,ti,kw OR 'casting':ab,ti,kw OR 'casts':ab,ti,kw OR 'traction':ab,ti,kw OR 'intramedullary':ab,ti,kw OR 'intra-medullary':ab,ti,kw OR 'nailing':ab,ti,kw OR 'titanium':ab,ti,kw) AND ('preschool child'/exp OR 'paediatric':ab,ti,kw OR 'pediatric':ab,ti,kw OR 'children':ab,ti,kw OR infan*:ab,ti,kw OR toddler*:ab,ti,kw OR minor:ab,ti,kw OR 'minors*':ab,ti,kw OR 'boy':ab,ti,kw OR 'boys':ab,ti,kw OR 'girl':ab,ti,kw OR 'girls':ab,ti,kw OR 'kid':ab,ti,kw OR 'kids':ab,ti,kw OR 'schoolchild*':ab,ti,kw OR 'juvenil*':ab,ti,kw OR 'prematur*':ab,ti,kw OR 'youth':ab,ti,kw OR 'youths':ab,ti,kw OR 'child'/exp OR 'child':ab,ti,kw) AND ('femoral shaft fracture*':ab,ti,kw OR 'femur fracture'/exp OR 'femur fracture*':ab,ti,kw) →2415 results

Three independent reviewers screened title and abstract using Rayyan QCRI. Subsequently, they screened full texts of selected articles. All articles on pediatric femoral shaft fractures comparing two or more treatments were potentially eligible. We included studies with a sample size with a mean age within 2–10 years. Randomized controlled trials (RCTs), cohort studies and observational studies were included. Reviews, case reports, comments and letters were excluded. Articles on treatment of open femur fractures were excluded. Also, articles with a follow-up of less than 3 months were excluded. Other exclusion criteria were absence of reported outcome or irrelevant outcome measures and non-English articles. Finally, articles selected for the systematic review were assessed for eligibility for the meta-analysis. Disagreements were resolved through discussion and decided on by the third reviewer.

### Data extraction

Data extraction was performed independently by three reviewers with the use of a predefined data extraction form. The following characteristics were extracted from the included studies: first author, year of publication, study design, number of included patients, length of follow-up, included age groups and relevant outcomes. Studies reporting on patient cohorts described in previously published articles were excluded or merged.

### Measurement of treatment outcome

The outcomes of interest were pre-determined, decided on by the senior author. Primary treatment outcomes were divided in radiological outcome and rehabilitation. Radiological outcome was assessed in terms of malunion (rate), angulation (degrees) and leg length discrepancy or shortening (cms). Rehabilitation was assessed in terms of length of hospital stay, time until walking with aids, time until independent ambulation and time until full weight bearing. Secondary treatment outcomes were complication rate and quality of life (QoL). Before data extraction, possible complications were categorized into mild and severe complications. Mild complications were defined as those that did not require operative treatment and would not cause future disability. Major complications were defined as those that led to unscheduled operative treatment, prolonged morbidity and/or disability. When the severity of a documented complication was unclear, it was decided upon through discussion. Regarding QoL, available literature was screened, but a lack of QoL specific outcome measures was noted. To still gain some insight in patient experience after treatment, we used patient/parent satisfaction as best available measure.

### Quality assessment

Risk of bias assessment was performed at study level, using The Cochrane Risk of Bias tool (RoB) for the assessment of risk of bias of randomized controlled trials. For observational studies a modification of this tool was used, in which *comparability of baseline characteristics* and *concurrency of cohorts* were added to the assessment. We assessed quality of evidence of the RCTs using the GRADE tool.

### Statistical analysis

Data were analyzed in October, 2020. As principle summary measures, mean differences (MD) were calculated for continuous outcomes and risk difference (RD) for dichotomous outcomes. When sufficient data were available confidence intervals were calculated. When SDs were missing they were calculated by use of the Cochrane SD calculator. All analyses were performed using random-effects models. We assessed statistical heterogeneity between studies by visual inspection of forest plots and *I*^2^ tests. The significance level for treatment effects was determined by the overall-effect *z* test. Potential publication bias was assessed by visual assessment of funnel plots. When both RCTs and observational studies were identified, the authors performed subgroup analysis and presented both results of the pooled RCTs and total results. Moreover, subgroup analysis was performed on children of 2–6 years of age whenever a minimum of two studies investigated an outcome in this age group. For these analyses, suitable sample sizes were defined as those with a mean age of in between 3 and 5 years. Statistical analyses were performed using Review Manager (RevMan 5).

## Results

**Fig. 1:**
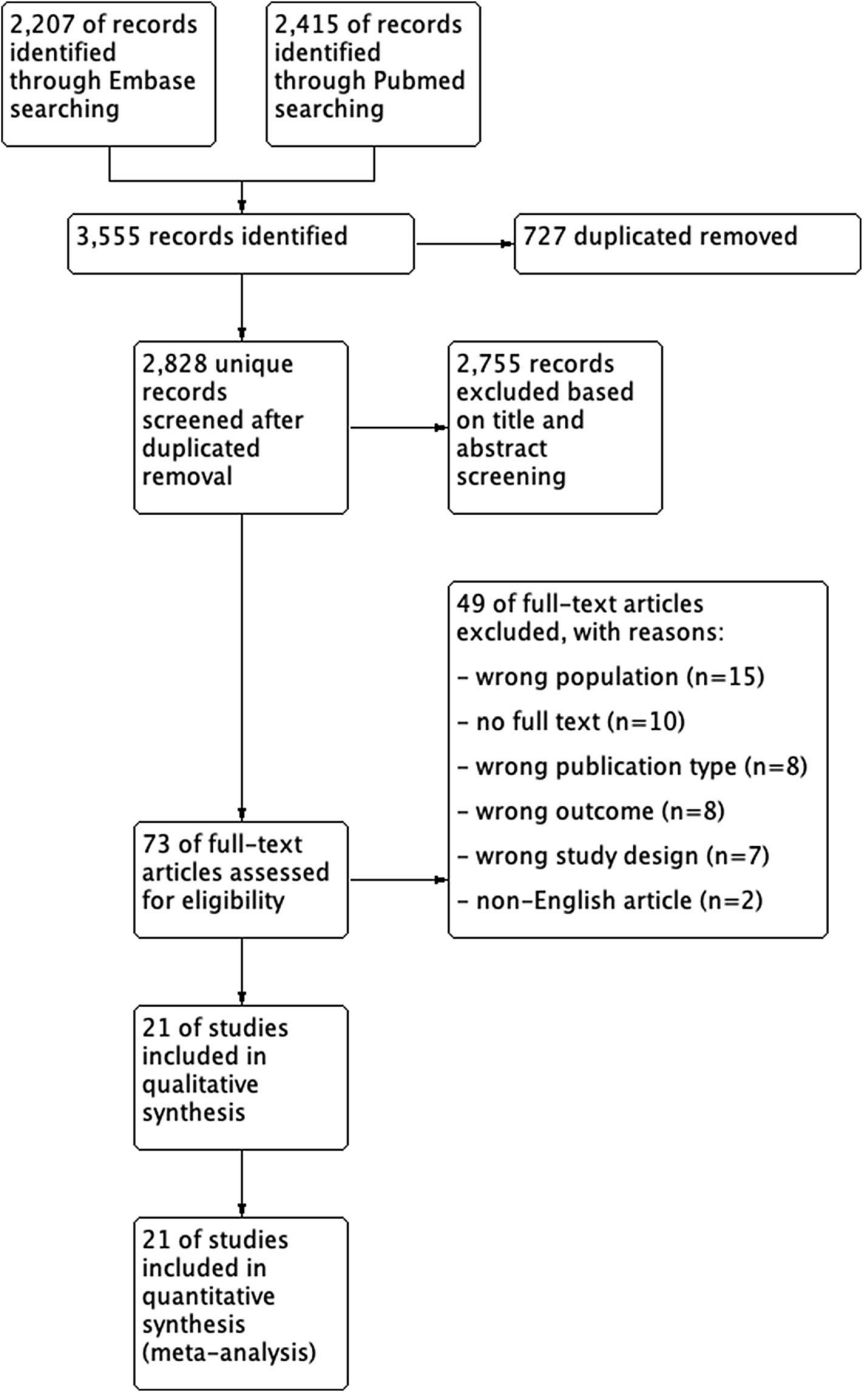
PRISMA flow diagram

A total of 2828 potentially relevant unique articles were retrieved and assessed for eligibility. Based on screening of titles and abstracts, 73 published studies were selected. No additional records were identified after backwards reference searching. The full text of the selected 73 articles was read for further selection. 52 articles were excluded, based on inappropriate study population, primary outcomes, study design, publication type or language. A total of 21 articles reporting on 1675 patients met all inclusion criteria and were finally included in this meta-analysis. Figure 1 presents a flow diagram depicting the stages of study selection and reasons for exclusion.

### Immediate spica cast versus intramedullary nail

Our search found seven unique articles that compared immediate casting and intramedullary nails (IMN) as treatment for femoral shaft fractures (Table 3). One article was an RCT [7] one article was a quasi-prospective comparative study [8] and five studies were retrospective comparative studies [9–13].

**Table 3:** Summary of included studies comparing treatment modalities

Immediate spica casting versus intramedullary nailing						
Study	Study design	Age group (mean)	Group size	Follow-up	Relevant outcomes	Remarks
Ramo et al. [13]	Retrospective cohort study	4–6 years (y) (Cast 4.7y, IMN 5.2y)	Cast: *n* = 158 IMN: *n* = 104	Cast 25 wks vs. IMN 44 wks, *P* < 0.001	Coronal angulation, sagittal angulation, shortening, complications	
Heffernan et al. [12]	Retrospective multicenter study	0–6y (Cast 3.2y, IMN 4.5y)	Cast: *n* = 141 IMN: *n* = 74	Cast 1.2 ± 1.5 y vs. IMN 3.7 ± 2.7 y, *P* < 0.001	Coronal angulation, sagittal angulation, length of hospital stay, leg length, time to rehabilitation,	
Ruhallah et al. [7]	RCT	3–12y (Cast 5.6y, IMN 6.92y)	Cast: *n* = 25 IMN: *n* = 25	Cast 16 m, IMN 17 m	Hospital stay, time to rehabilitation, Flynn’s grading, complications	Was the only study to use Rush pins instead of TEN in the IMN group
Say et al.[10]	Retrospective comparative study	6–12y (Cast 6.4y, IMN 9.8y)	Cast: *n* = 20 IMN: *n* = 22	Cast 14.3 ± 6 m, IMN 12.6 ± 5.2 m	Hospital stay, malalignment, walking with aids, complications	
Assaghir et al. [11]	Retrospective comparative study	2–6y (Cast 4.1y, IMN 4.9y)	Cast: *n* = 52 IMN: *n* = 52	Cast 41 ± 3.9 m, IMN 40.3 ± 3.5 m *P* = 0.301	Leg shortening, coronal and sagittal angulation, hospital stay, weight bearing, rotation, time to rehabilitation, complications	
Jauquier et al. [9]	Retrospective comparative study	1–4y (Cast 26 months (m), IMN 28,4 m)	Cast: *n* = 19 IMN: *n* = 27	Cast 114 m IMN 16.5 m	Malunion, leg length discrepancy, hospitalization, full weight bearing, immobilization, complications	
Saseendar et al. [8]	Quasi prospective comparative study	5–15 y (Cast: 9.25 y, IMN: 10 y)	Cast: *n* = 16 IMN: *n* = 16	Cast min. 1 y, IMN 12–18 m	Coronal and sagittal angulation, rotational malalignment, LLD, weight bearing	Cast group was treated with a Steinmann pin within the cast
Traction and subsequent spica casting (TSC) versus intramedullary nailing						
Soleimanpour et al. [17]	RCT	6–12y (TSC 8.33y, IMN 8.73y)	TSC: *n* = 60 IMN: *n* = 60	1 y	Mean time spent in hospital, walking with aids, independent walking, LLD, angulation	
Nascimento et al. [20]	Retrospective comparative study	5–13 y (TSC 8y, IMN 9.6y)	TSC: *n* = 30 IMN: *n* = 30	TSC: 59.0 m IMN : 35.4 m	Hospitalization, shortening, angulation, weight bearing	There was no mention of duration of traction
Shemshaki et al. [16]	RCT	6–12 y (TSC 6.5y, IMN 7.1y)	TSC: *n* = 23 IMN: *n* = 23	24 w	Length of hospital stay, alignment, rotation, time to walking with aids and independent walking	
Hsu et al. [18]	RCT	5–12y (TSC, 7.3y, IMN 8.7y)	TSC: *n* = 25 IMN: *n* = 26	Min. 12 w	Hospital stay, angulations,	
Mehdinasab et al. [15]	RCT	6–11y (TSC 7.2y, IMN 8.1y)	TSC: *n* = 30 IMN: *n* = 36	6 m	Duration of hospital stays, ambulation malrotation, shortening	IMN group received cast after IMN. Mehdinasab et al. described a randomization process in their methods, the two treatment groups aren’t even in patient number. This difference is not explained, and loss to follow-up does not compensate for this difference
Flynn et al. [19]	Prospective cohort study	6–16y (TSC 8.7y, IMN 10.2y)	TSC: *n* = 35 IMN: *n* = 48	Min. 1 y	Alignment, LLD, angulation, hospitalization, walking independently, walk with support	
Song et al. [21]	Retrospective comparative study	4–11y (TSC 6y, 11 m, IMN 7y,1 m)	TSC: *n* = 24 IMN: *n* = 27	TSC: 59.2 m, IMN: 30.3 m	Malalignment, angulation, LLD, Flynn criteria, weight bearing	Song et al. converted to spica cast as soon as callus was seen on X-ray scans
Buechsensuetz et al. [22]	Retrospective comparative study	4–14 (TSC 6y, 9 m, IMN 8 y years 7 m)	TSC: *n* = 29 IMN: *n* = 42	2 y, 3 m	Flynn criteria, weight bearing, malunion	
Traction with subsequent spica casting (TSC) versus immediate casting						
Younis et al. [28]	Retrospective comparative study	0-6y (TSC: 3 y Cast: 2 y 5,5 m)	TSC: *n* = 20 Cast: *n* = 24	22.8 m (range: 9–56 m)	weight bearing, activities, length of hospital stay, LLD, angulation, complications	Casting after at least 48 h of traction vs casting within 48 h
D’Ollonne et al. [26]	Retrospective comparative study	2–6y (TSC: 3.2 y Cast: 2.4 y)	TSC: *n* = 14 Cast: *n* = 21	38.5 m (24–96 m)	Malalignment, weight bearing, malunion, LLD, angulation, complications	Casting after 3 weeks of traction compared to immediate casting
Siddiqui et al. [23]	RCT	3–10y (TSC: 7.8 y Cast: 7.6 y)	TSC: *n* = 21 Cast: *n* = 21	Not reported	Satisfactory outcomes (Shortening, angulation, complications)	Casting after 3 weeks of traction compared to immediate casting
Yandow et al. [27]	Retrospective comparative study	0–15y (TSC: 5.2 y Cast: 3.7 y)	TSC: *n* = 55 Cast: *n* = 33	Mean: 8.9y. Range 4–20 y	Angulation, LLD, complications	Casting after at least 48 h of traction vs casting within 48 h
Curtis et al. [24]	Prospective cohort study	2–10y (TSC: 6.3y Cast: 5.6y)	TSC: *n* = 21 Cast: *n* = 70	Mean: tr + cast: 78 m, cast: 44 m, min. 2 y	Malalignment, LLD, complications	Used the Pontoon spica cast in both groups
Henderson et al. [25]	Prospective cohort study	0–10y (5.3 y)	TSC: *n* = 50 Cast: *n* = 26	Not reported	Days of hospital stay, complications	Casting after early callus formation with traction compared to immediate casting

*Y* years, *m* months, *w* weeks, *TSC* traction with subsequent casting, *IMN* intramedullary nails, *LLD* leg length discrepancy

### RCT and quality of evidence

**Fig. 2:**
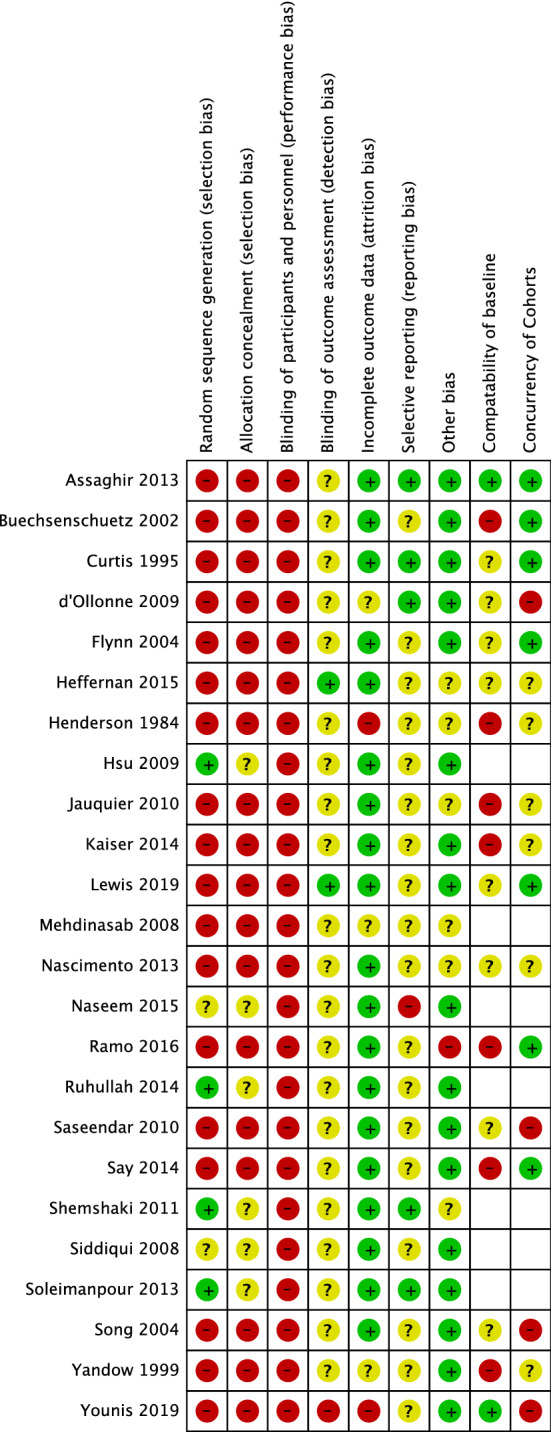
Risk of bias assessment (1)

**Fig. 3:**
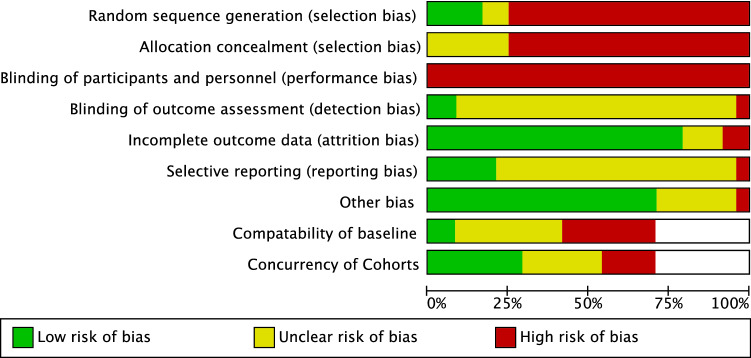
Risk of bias assessment (2)

In their RCT, Ruhallah et al. [7] investigated the age group of 3–12 years old and compared treatment with Rush pins with immediate spica casting. The trial was at some risk of bias (Figs. 2, 3). Quality of evidence was assessed by use of the GRADE tool. As this was the only RCT that investigated these two interventions and its low-to-moderate risk of bias, the quality of evidence based on this RCT was estimated to be ‘very low’.

### Observational studies

Seven observational studies were assessed for risk of bias (Figs. 2, 3). One study was at lower risk of bias [11]. The remaining articles [8–10, 12, 13] were all assessed to have a high risk of bias.

### Radiological outcome

**Fig. 4:**
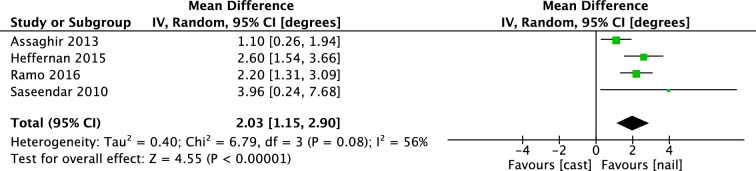
Forest plot: mean coronal angulation in cast group versus nail group

In the RCT of Ruhallah et al., radiological outcome was presented by use of Flynn’s criteria [14]. In the IMN group, 88% of the fractures recovered with malalignment under 5°, compared to 20% in the cast group. Of the remaining patients treated with spica cast 38% had malalignment of 5–10° and 42% over 10°. In the intramedullary nailing group, 8% had malalignment of 5–10° and 4% over 10°. No measure of dispersion or P value was reported. Four observational studies conveyed malalignment in mean coronal and sagittal angulation, investigating a total of 611 patients. As displayed in Fig. 4, the mean difference of coronal angulation (IV, Random, 95% CI [degrees]) was 2.03 degrees [1.15, 2.90] in favor of IMN. Subgroup analysis of children aged 2–6 years showed a mean difference of 1.93 degrees [1.03, 1.82].

**Fig. 5:**
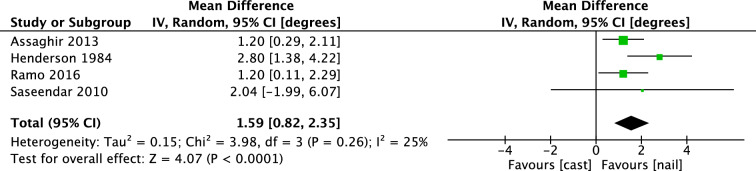
Forest plot: mean sagittal angulation in cast group versus nail group

The mean difference of sagittal angulation (IV, Random, 95% CI [degrees]) was 1.59 degrees [0.82, 2.35] in favor of IMN (Fig. 5). Subgroup analysis of children aged 2–6 years showed a mean difference of 1.61 degrees [0.70, 2.51].

Six studies included ‘leg length discrepancy’ (LLD) in their investigation. Ruhallah et al. again conveyed LLD as part of Flynn’s criteria assessment. Results are shown in Table 4.

**Table 4:** Comparison of leg length discrepancy in Ruhallah et al.

Group	< 1 cm	1–2 cm	> 2 cm
Cast	11 (45%)	6 (25%)	7 (30%)
IMN	22 (88%)	3 (12%)	0 (0%)

*IMN* intramedullary nailing

**Fig. 6:**
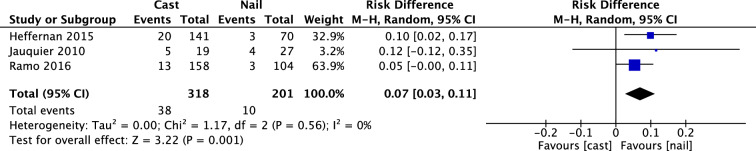
Forest plot: risk difference of leg length discrepancy in cast group versus nail group

Three other studies reported the occurrence rate of LLD. The mean risk difference (M-H, Random, 95% CI) was 0.07 [0.03, 0.11] in favor of IMN (Fig. 6). All three studies investigated children of 2–6 years old. Finally, two observational studies reported mean LLD. Their pooled mean difference was not significant: 0.39 cm [-0.16, 0.94]. There was no visual asymmetry in the funnel plots of radiological outcomes, indicating no evidence of publication bias.

### Rehabilitation

**Fig. 7:**
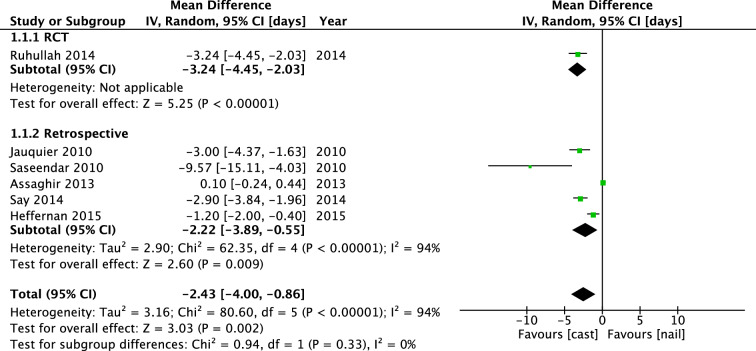
Forest plot: mean days of hospital stay in cast group versus nail group

Six studies investigated length of hospital stay in a total of 489 patients. The one RCT of Ruhallah favored spica casting: the mean difference (IV, Random, 95% CI [days]) was − 3.24 days [− 4.45, − 2.03]. The pooled mean difference was − 0.68 days [− 0.96, − 0.39] (Fig. 7).

**Fig. 8:**
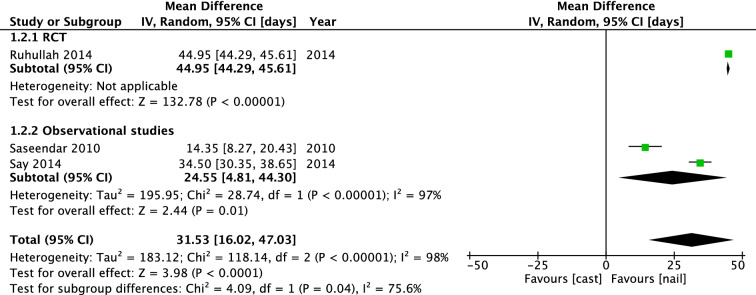
Forest plot: mean days until walking with aids in cast group versus nail group

In 2–6-year old children, the mean difference was -1.75 days [− 3.38, − 0.12], favoring immediate casting. Three studies included 124 patients to investigate time until walking with aids. In the RCT of Ruhallah et al., the IMN group started walking with aids significantly earlier with a mean difference of 44.95 [44.29, 45.61] days. Pooled mean difference (IV, Random, 95% CI [days]) was 31.53 days [16.02, 47.03], in favor of the IMN group (Fig. 8).

**Fig. 9:**
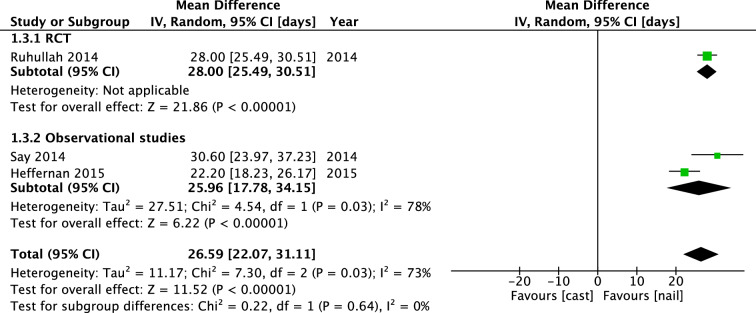
Forest plot: mean days until independent ambulation in cast group versus nail group

**Fig. 10:**
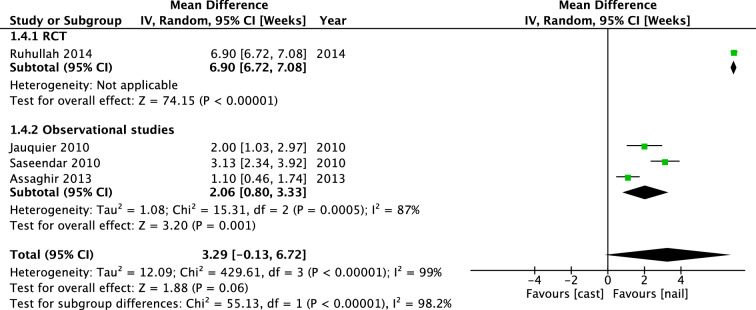
Forest plot: mean days until full weight bearing in cast group versus nail group

In a total of 307 patients in three studies, time until independent ambulation was investigated. Ruhallah et al. found a mean difference of 28.00 days [25.49, 30.51] in favor of the IMN group. Pooled mean difference (IV, Random, 95% CI [days]) was 26.59 days [22.07, 31.11] (Fig. 9). Subgroup analysis of children aged 2–6 years showed a mean difference of 25.20 days [19.34, 31.07]. Weeks until full weight bearing was investigated in 4 studies, in a total of 232 patients. In the RCT of Ruhallah et al., patients who received treatment with intramedullary nailing were bearing full weight significantly earlier with a mean difference of 6.90 days [6.72, 7.08]. The pooled mean difference (IV, Random, 95% CI [weeks]) of all four studies was not significant: 3.29 weeks [− 0.13, 6.72] (Fig. 10). Subgroup analysis of children aged 2–6 years showed a mean difference of 3.35 weeks [− 1.04, 7.73]. There was no visual asymmetry in the funnel plots of reported outcomes, indicating no evidence of publication bias.

## Traction and cast versus intramedullary nail

### Search results

Our search yielded eight unique articles that compared traction and cast with intramedullary nails as treatment for femoral shaft fractures (Table 3). Four articles were RCTs [15–18], one was a prospective cohort study [19] and three studies were retrospective comparative studies [20–22]

### RCTs and quality of evidence

Soleimanpour et al. investigated a population of 6–12 years old and compared 3 weeks of traction and subsequent spica casting (TSC) with titanium elastic nailing. Shemshaki et al. investigated patients of 6–12 years old. Children in the TSC group were treated with 3 weeks of skeletal traction. The IMN group was treated by titanium elastic nails. Hsu et al. investigated 5–2-year-old patients in a resource-limited setting. Patients in the conservative group were treated simultaneously with traction and spica cast. The IMN group was treated by TEN.

Mehdinasab et al. enrolled patients of 5–11 years old with a follow-up of 6 months. The TSC received skeletal traction and a spica cast was applied as soon as there was a mitigation of pain. IMN was performed with TEN.

All four RCTs were assessed on risk of bias by use of the Cochrane Risk of Bias tool. All studies had some risk of bias. The study of Mehdinasab et al. was assessed to have a high risk of bias (Figs. 2, 3). The quality of evidence was assessed by use of the GRADE tool. Because of the risk of bias, the high mean age in all articles and the conflicting results of these studies, quality of evidence was assessed to be ‘low’.

### Observational studies

Our search yielded four non-RCT’s. Because of the observational study design, all studies had a high risk of selection bias and performance bias. All four studies were at a relatively high risk of bias (Figs. 2, 3).

### Radiological outcome

**Fig. 11:**
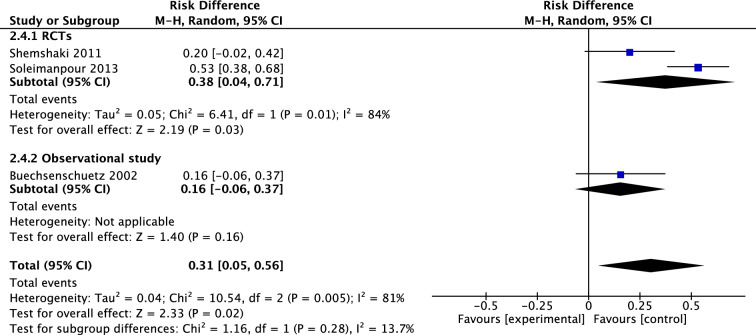
Forest plot: risk difference of malunion in TSC group versus nail group

**Fig. 12:**
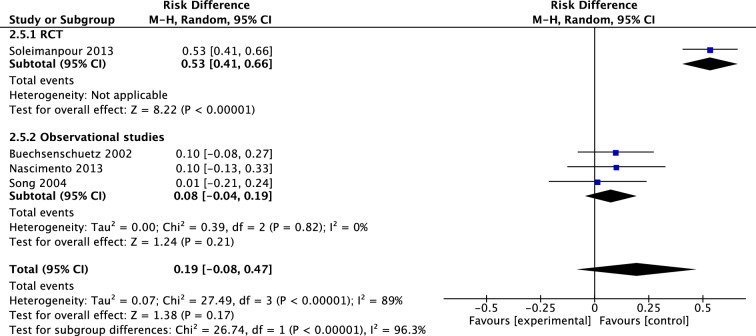
Forest plot: risk difference of leg length discrepancy in TSC group versus nail group

Three studies compared rate of malunion in femoral fractures treated with immediate cast and with intramedullary nails, in a total of 218 patients. The RCT’s of Shemshaki et al. and Soleimanpour et al. had a mean risk difference (M-H, Random, 95% CI) of 0.38 [0.04, 0.71] in favor of IMN. Pooled mean difference (M-H, Random, 95% CI) of all three studies was 0.31 [0.05, 0.56] in favor of IMN (Fig. 11). Four studies investigated 299 patients for the occurrence of limb length discrepancy after treatment. The only RCT of Soleimanpour et al. found a risk difference of 0.53 [0.41, 0.66]. The pooled mean risk difference (M-H, Random, 95% CI) of all four studies was 0.19 [− 0.08, 0.47] in favor of IMN, however, not significant (Fig. 12). Finally, four studies reported on angulation after treatment of femoral fractures in both groups. The RCT of Soleimanpour found a coronal angulation in 26.6% in the TSC group compared to 13.3% in the IMN group, and sagittal angulation in, respectively, 20% and 6.7%. There was no measure of significance.

Hsu et al. and Song et al. reported conflicting results for coronal angulation and sagittal angulation. Pooled mean difference (IV, Random, 95% CI) of coronal angulation was 0.46 degrees [− 1.34, 2.27] in favor of IMN. Pooled mean difference (IV, Random, 95% CI) of sagittal angulation was 2.88 degrees [− 0.65, 6.41] in favor of IMN. There was no visual asymmetry in the funnel plots of these results, indicating no evidence of publication bias.

### Rehabilitation

**Fig. 13:**
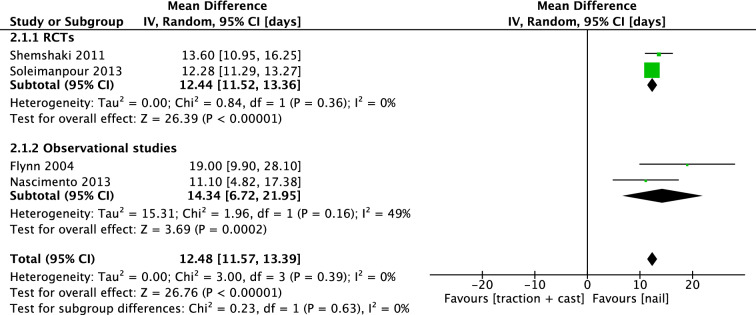
Forest plot: mean days of hospital stay in TSC group versus nail group

Seven studies reported mean days of hospital stay. Mehdinasab et al. and Buechsensuetz et al. reported no measure of dispersion. Hsu et al.’s results could only be generalized in a resource-limited setting and this did not apply to our research question. Therefore, we were able to include four studies in the analysis. Shemshaki and Soleimanpour had a combined mean difference (IV, Random, 95% CI) of 12.44 days [11.52, 13.36], in favor of IMN. The pooled mean difference (IV, Random, 95% CI) was 12.48 days [11.57, 13.39] in favor of IMN (Fig. 13).

**Fig. 14:**
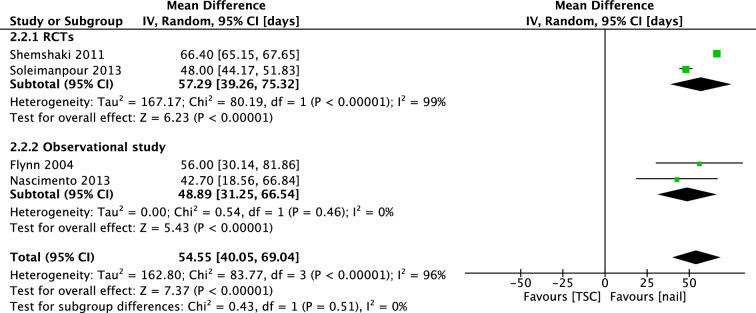
Forest plot: mean days until walking with aids in TSC group versus nail group

**Fig. 15:**
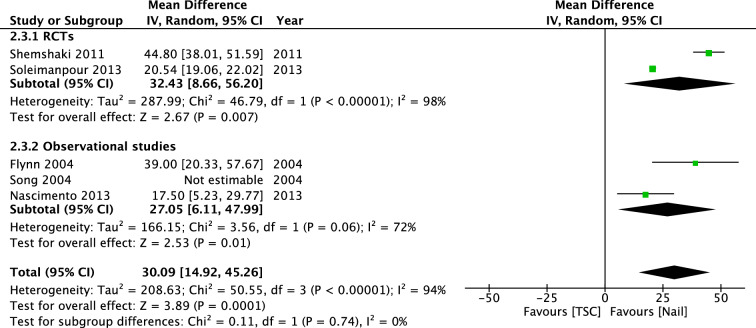
Forest plot: mean days until full weight bearing in TSC group versus nail group

Four studies investigated days until walking with aids. Two RCT’s (Shemshaki; Soleimanpour) found superior results for IMN, with a mean difference of 57.29 days [39.26, 75.32]. The pooled mean difference (IV, Random, 95% CI) of the four studies was 54.55 days [40.05, 69.04] (Fig. 14). Five studies reported days until full weight bearing after treatment. However, because Song et al. reported no measure of dispersion, we were able to include four studies in the meta-analysis. Shemshaki et al. and Soleimanpour et al. reported earlier full weight bearing in the IMN group, with a mean difference (IV, Random, 95% CI) of 32.43 days [8.66, 56.20]. Pooled mean difference (IV, Random, 95% CI) was 27.05 days [6.11, 47, 99] (Fig. 15). There was no visual asymmetry in the funnel plots of these outcomes, indicating no evidence of publication bias.

## Traction and cast versus cast

### Search results

Our search yielded six unique articles that compared traction and subsequent casting with immediate spica casting (Table 3). We found one RCT [23], two prospective cohort studies [24, 25] and three retrospective comparative studies [26–28].

### RCT and quality of evidence

Siddiqui et al. investigated the age group of 3–10 years old and compared immediate spica casting to traction with Thomas splint and subsequent casting after 3–4 weeks of soft callus formation.

We assessed the RCT of Siddiqui et al. on risk of bias by use of the Cochrane Risk of Bias tool (Figs. 2, 3). Quality of evidence was assessed by use of the GRADE tool. Reflecting this article to be the only RCT, the mean age in both groups to be higher than 2–6 years old and the moderate risk of bias, the quality of evidence based on this RCT was estimated to be ‘very low’.

### Observational studies

We yielded four observational studies. All studies were assessed on risk of bias (Figs. 2, 3). Curtis et al. had a relatively low risk of bias, while the other three studies were assessed to have a high risk of bias.

### Radiological outcome

**Fig. 16:**
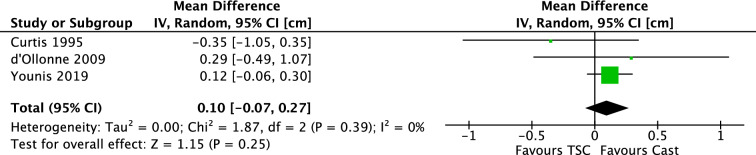
Forest plot: mean leg length discrepancy in TSC group versus cast group

Siddiqui et al. published an RCT comparing these two treatments. Results were described as either satisfactory or unsatisfactory. Fractures with shortening of more than 2 cm’s, coronal angulation of more than 15 degrees, sagittal angulation of more than 20 degrees or complications needing change in management, were categorized as unsatisfactory. The TSC group scored 5% unsatisfactory outcome compared to 19% in the cast group. In 3 studies, LLD was investigated in a total of 170 patients. The mean difference (IV, Random, 95% CI) was 0.10 cm [− 0.07, 0.27]. Subgroup analysis of children of 2–6 years old was performed, yielding a mean difference of 0.13 cm [− 0.05 m 0.30]. Results are displayed in Fig. 16. There was no visual asymmetry in the funnel plots of reported outcomes, indicating no evidence of publication bias. Three studies included coronal and sagittal angulation as outcome. In most studies, no mention of dispersion was reported.

The results are displayed in Table 5.

**Table 5:** Mean angulation in patients treated with TSC versus immediate spica cast

Study	Coronal angulation			Sagittal angulation		
	TSC	Cast	Significance	TSC	Cast	Significance
Yandow et al	1.0	1.0	Not reported	11.5	8.7	Not reported
Curtis et al	6.5	3	Not reported	2	2	Not reported
D’Ollonne et al	2.1	3.2	*p* = 0.625	2.2	3.2	n.s

The numbers displayed in the table are degrees of angulation

*TSC* traction and subsequent casting

### Rehabilitation

**Fig. 17:**
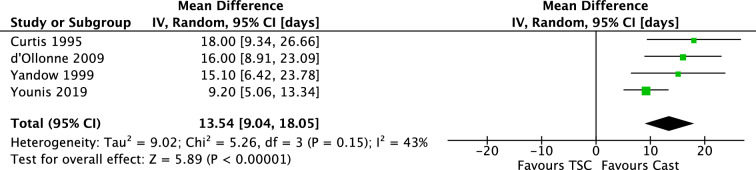
Forest plot: mean days of hospital stay in TSC group versus cast group

Four studies found the length-of-hospital stay to be significantly less in the immediate spica casting group. The total mean difference (IV, Random, 95% CI) was 13.54 days [9.04, 18.05] (Fig. 17). Mean difference in children aged 2–6 years was 12.44 [7.67, 17.22]. There was no visual asymmetry in the funnel plots, indicating no evidence of publication bias.

In terms of time to full weight bearing, D’Ollonne et al. reported a mean difference (IV, Random, 95% CI) of 14.00 days [7.44, 20.56] in favor of the cast group.

## Complications

Table 6 provides an overview of reported complication rates. The mean complication rate in the TSC group was 18.3%, compared to 15.9% in the nailing group and 14.5% in the spica cast group. The rate of major complications was highest in the TSC group (5.9%), followed by the nailing group (5.2%) and the cast group (2.3%). For treatment with TSC and immediate spica cast, reported minor complications were similar and consisted of skin complications as pressure sores and skin irritation [7, 9, 11–13, 19, 22, 24, 26–28], mild loss of reduction problems requiring cast adjustment [15, 16, 19, 24, 26, 28], mesenteric artery syndrome [25, 27], superficial infection [16, 18], knee stiffness [19], loosening of spica [7], painless limping, out-toeing [11] and temporary peroneal nerve palsies [27]. Reported minor complications in the IMN group were nail end irritation [7, 9, 11–13, 15, 18, 20–22], mild nail exteriorization [9, 11, 18, 20], superficial infection [15, 21, 22], mild loss of reduction [15, 20] and rotation limitation [13]. Major complications in the TSC group were severe loss of reduction [15, 26], or sepsis [27], fat emboli syndrome [27], postcast contralateral limb fracturing [24] and refracture [22].

**Table 6:** Rate of complications in studies comparing TSC to intramedullary nails

TSC	Number of patients	Total reported complications	Minor complications	Major complications
Nascimento [20]	*n* = 30	3 *(10%)*	1 *(3.3%)*	2 *(6.7%)*
Shemshaki [16]	*n* = 23	3 *(13%)*	0 (0%)	3 *(13%)*
Hsu [18]	*n* = 25	2 *(8%)*	2 *(8%)*	0 *(0%)*
Mehdinasab [15]	*n* = 30	7 *(23.3%)*	4 *(13.3%)*	3 *(10%)*
Flynn [19]	*n* = 35	12 *(34%)*	6 *(17%)*	6 *(17%)*
Song [21]	*n* = 24	2 *(8,3%)*	2 *(8.3%)*	0 *(0%)*
Buechsensuetz [22]	*n* = 29	10 *(34%)*	10 *(34%)*	3 *(10%)*
Younis et al. [28]	*n* = 20	4 *(16.6%)*	4 *(16.6%)*	0 *(0%)*
D’Ollonne [26]	*n* = 14	3 *(21.4%)*	2 *(14.3%)*	1 *(7.1%)*
Yandow [27]	*n* = 55	5 (*9.1%)*	3 *(5.5%)*	2 *(3.6%)*
Curtis [24]	*n* = 21	10 *(48%)*	8 *(38%)*	2 *(10%)*
Henderson [25]	*n* = 50	8 *(16%)*	8 (*16%)*	0 *(0%)*
**Means**	***n* = 376**	**69 *(18.3%)***	**50 *(13.3%)***	**22*(5.9%)***

IMN	Number of patients	Total reported complications	Minor complications	Major complications
Nascimento [20]	*n* = 30	3 *(10%)*	2 *(6.7%)*	1 *(3.3%)*
Shemshaki [16]	*n* = 23	3 *(13%)*	0 (0%)	3 *(13%)*
Hsu [18]	*n* = 26	3 *(11.5%)*	3 *(11.5%)*	0 *(0%)*
Mehdinasab [15]	*n* = 36	6 *(16.7%)*	6 *(16.7%)*	0 (*0%)*
Flynn [19]	*n* = 48	10 *(21%)*	6 *(12.5%)*	4 *(8.3%)*
Song [21]	*n* = 27	4 *(14.8%)*	1 *(3.7%)*	3 *(11.1%)*
Buechsensuetz [22]	*n* = 42	9 *(22%)*	7 *(17%)*	2 *(5%)*
Ramo [13]	*n* = 104	17 *(16.3%)*	9 *(8.7%)*	8 *(7.7%)*
Ruhallah [7]	*n* = 25	2 *(8%)*	2 *(8%)*	0 *(0%)*
Assaghir [11]	*n* = 52	10 *(19%)*	9 *(17%)*	1 *(2%)*
Jauquier [9]	*n* = 27	3 *(11%)*	2 (*7.4%)*	1 *(3.7%)*
**Means**	***n* = 440**	**70 *(15.9%)***	**47 *(10.7%)***	**23 *(5.2%)***

Cast	Number of patients	Total reported complications	Minor complications	Major complications
Ramo [13]	*n* = 158	12 *(7.6%)*	3 *(1.9%)*	9 *(5.7%)*
Ruhallah [7]	*n* = 25	4 *(17%)*	4 *(17%)*	0 *(0%)*
Assaghir [11]	*n* = 52	20 *(38%)*	20 *(38%)*	0 *(0%)*
Jauquier [9]	*n* = 19	2 *(10.5%)*	2 *(10.5%)*	0 *(0%)*
Younis et al. 2019[28]	*n* = 24	6 *(30%)*	6 *(30%)*	0 *(0%)*
D’Ollonne [26]	*n* = 21	3 *(14.3%)*	3 *(14.3%)*	0 *(0%)*
Yandow [27]	*n* = 33	1 *(3.0%)*	1 *(3%)*	0 *(0%)*
Curtis [24]	*n* = 70	11 *(15.7%)*	11 *(15.7%)*	0 *(0%)*
Henderson [25]	*n* = 26	3 *(11.5%)*	2 *(7.7%)*	1 *(3.8%)*
**Means**	***n* = 428**	**62 *(14.5%)***	**52 *(12.1%)***	**10 *(2.3%)***

*RCT* randomized controlled trial, *TSC* traction and subsequent casting, *IMN* intramedullary nailing, *LLD* leg length discrepancy, *y* years, *m* months, *y* years, *m* months, *n* number

For IMN, reported major complications were implant failure [9, 13, 19, 21], implant infection [11, 13, 19], pulmonal embolus [22] and refracture [19].

The major complications in the cast group were loss of reduction [13] and failure of the pin that was used for traction within the cast [25].

## Quality of life: patient satisfaction

Four studies investigated patient satisfaction after treatment. Because there was no general assessment method, we were unable to pool results. All studies reported higher patient satisfaction in the IMN groups. Buechsensuetz et al. contacted patients’ parents and found that 93% of the IMN group would ‘definitely’ choose the same treatment again, compared to only 6% of the TSC group (*p* < 0.001) [22]. Shemshaki et al. found that 100% of parents of IMN patients rated treatment outcome as either ‘Good’ or ‘Excellent’, compared to 74.1% of the TSC group (*p* = 0.003) [16]. Mehdinasab et al. reported that patients who received IMN were more satisfied without a description of assessment methods or further depiction of results [15].

## Discussion

### Results and previous literature

The most important findings of this study are that for femur fractures in children of 2–10 years, treatment with intramedullary nails was associated with significantly lower rates of malunion and LLD, lower means of angulation and shortening and earlier achievement of rehabilitation milestones compared to treatment with both immediate spica casting as TSC. Moreover, subgroup analysis of children of 2–6 years old yielded similar results. Therefore, this study demonstrates a tendency to intramedullary nailing as the preferred treatment of femur fractures in children ages 2–6 years. Compared to TSC, immediate spica casting led to earlier achievement of rehabilitation milestones but did not significantly differ in other outcomes. Both severe complication rate and total complication rate were highest in the TSC group and lowest in the spica cast group. There was great variation between studies in what were considered complications. Moreover, the nature of complications differed per intervention. Therefore, results should be interpreted with caution. Nevertheless, the nature of these complications is in line with existing literature: in traction and spica casting, the most common adverse effects seen are skin breakdown and other skin complications [29–32]. Skin traction carries the risk of pressure sores, while skeletal traction can lead to bone damage [33]. Other complications for spica casting include compartment syndrome and superior mesenteric artery syndrome [34, 35]. Intramedullary nailing comes with general risks related to anesthesia and surgery risks like wound infection [7, 16, 36]. Also, nail end irritation and nail exteriorization have been documented as complications [9, 37]. Moreover, this surgical treatment requires a second procedure for elective implant removal, which again comes with general anesthesia and surgery risks [13, 38, 39].

In recent meta-analysis in 2018, Imam et al. compared spica casting to intramedullary nailing and reported a significant statistical difference favoring IMN in terms of duration of hospital stay, time to independent walking and patient satisfaction. Similarly, rates of malunion and angulation and duration of union significantly favored the IMN group. Therefore, they recommended the use of IMN fixation, which is, to some extent, in line with this study’s conclusions. However, Imam et al. included children below 16 years old in their review. Moreover, unlike this study, they did not perform subgroup analysis and no distinction between immediate casting and casting after traction was made [29].

From the early 00’s onward, significant changes in the approach of femoral shaft fractures have been presented. Particularly in school aged children (6–12 years old), surgical intervention has become the preferred treatment especially because of a short mean hospital stay and early return to daily activities [40]. Among other similar narrative reviews (Gardner [40], Flynn [41]), Heyworth et al. provided a management strategy for pediatric diaphyseal femur shaft fractures in 2012, prescribing immediate spica casting for children of 2–5 years old, and surgical intervention in children of 6–12 years old. Pavlik bandage and traction should be reserved for the youngest children, although traction is recommended as temporary option as well, until definitive treatment follows [39]. These guidelines have been roughly followed in general practice throughout the years. However, in 2019, Alluri et al. identified temporal trends in the management of femoral shaft fractures in 4- and 5-year-old children, finding that between 1997 and 2012, surgical fixation has increased with 35% for 4-year olds and 58% in 5-year olds. They, therefore, stated that the lower age limit for surgical management of these fractures was decreasing [42]. This trend was not supported by available evidence, as in 2014 Madhuri et al. conducted a systematic review comparing all treatment modalities for pediatric femoral shaft fractures. They concluded that based on their analysis, insufficient evidence existed to provide reliable recommendations on the matter [6].

Although studies investigating external fixation were not included in our analysis, there are two systematic reviews [43, 44] comparing elastic intramedullary nailing to external fixation for the treatment of pediatric femoral shaft fractures. Both authors concluded that although high-quality studies are limited, IMN leads to fewer complications and is the preferred approach for femoral shaft fractures in children. As plate fixation in general is not considered a treatment modality for this age group, we decided not to include this in our study either. Nevertheless, several studies found that IMN has better outcomes than plate fixation at young age [45–50].

### Limitations

In the meta-analyses comparing immediate casting and TSC to IMN, heterogeneity was high in several outcomes. Because of this, total mean differences may appear less reliable. However, in none of these outcomes there were conflicting results. Still, those results should be interpreted with caution.

Second, in studies investigating TSC, there was a variety in how long traction was continued until spica cast was applied. In one study, there was no mention of duration of traction [20], and in one study, a cast was applied in the IMN group as well [15]. This might have influenced results.

Another potential limitation is the exclusion of non-English-language studies, which might have caused bias. However, because selection was performed manually instead of by filter, the authors do not expect to have excluded relevant articles.

Cost of treatment was not included as outcome measure. Although a point of interest, the authors believed that only when all other outcomes would be equal, costs should be considered as outcome measure to determine superior treatment.

Lastly, unfortunately, we were not able to distinguish between fracture types in our analysis.

Finally, we acknowledge that a meta-analysis can only be as good as the primary studies that are included in the meta-analysis. The results of this study were limited by the limitations of the single studies. Therefore, we unfortunately were not able to distinguish between fracture types in our analysis. Also, we were unable to pool results regarding quality of life.

Still, this is the most extensive systematic review to date, and the first to compare several types of conservative treatment and surgical treatment of femoral shaft fractures in this specific age group.

## Conclusion

Although several studies have been published on the treatment of femoral shaft fractures in children, choice of treatment in children of 2–10 years old can often be challenging. Especially in the age group of 2–6 years old, no consensus on treatment has been reached. This systematic review and meta-analysis revealed a lack of high-quality RCTs on the subject to fill this knowledge gap, but shows a clear tendency to treatment with elastic intramedullary nails, both in general as in 2–6-year olds. While intramedullary nailing requires subsequent implant removal which comes with additional anesthesia and surgery risks, it appears to lead to superior radiological outcomes and significantly faster rehabilitation and ambulation. While in children older than 6 years old, it has been adopted as preferred treatment modality, this review justifies the use of IMN in younger children as well. Nevertheless, to provide a definitive recommendation on future clinical practices, high-quality evidence is necessary.
